# Comparison of the Effects of Dibutyl and Monobutyl Phthalates on the Steroidogenesis of Rat Immature Leydig Cells

**DOI:** 10.1155/2016/1376526

**Published:** 2016-04-11

**Authors:** Linxi Li, Xiaomin Chen, Guoxin Hu, Sicong Wang, Renai Xu, Qiqi Zhu, Xiaoheng Li, Mingcang Wang, Qing-Quan Lian, Ren-Shan Ge

**Affiliations:** ^1^Center of Scientific Research, The Second Affiliated Hospital & Yuying Children's Hospital, Wenzhou Medical University, Wenzhou, Zhejiang 325027, China; ^2^Department of Anesthiology, The Second Affiliated Hospital & Yuying Children's Hospital, Wenzhou Medical University, Wenzhou, Zhejiang 325027, China; ^3^School of Pharmacy, Wenzhou Medical University, Wenzhou, Zhejiang 325035, China; ^4^Taizhou Hospital of Zhejiang Province, Taizhou 317000, China

## Abstract

Dibutyl phthalate (DBP) is a widely used synthetic phthalic diester and monobutyl phthalate (MBP) is its main metabolite. DBP can be released into the environment and potentially disrupting mammalian male reproductive endocrine system. However, the potencies of DBP and MBP to inhibit Leydig cell steroidogenesis and their possible mechanisms are not clear. Immature Leydig cells isolated from rats were cultured with 0.05–50 *μ*M DBP or MBP for 3 h in combination with testosterone synthesis regulator or intermediate. The concentrations of 5*α*-androstanediol and testosterone in the media were measured, and the mRNA levels of the androgen biosynthetic genes were detected by qPCR. The direct actions of DBP or MBP on CYP11A1, CYP17A1, SRD5A1, and AKR1C14 activities were measured. MBP inhibited androgen production by the immature Leydig cell at as low as 50 nM, while 50 *μ*M was required for DBP to suppress its androgen production. MBP mainly downregulated* Cyp11a1 *and* Hsd3b1* expression levels at 50 nM. However, 50 *μ*M DBP downregulated* Star*,* Hsd3b1*, and* Hsd17b3* expression levels and directly inhibited CYP11A1 and CYP17A1 activities. In conclusion, DBP is metabolized to more potent inhibitor MBP that downregulated the expression levels of some androgen biosynthetic enzymes.

## 1. Introduction

Dibutyl phthalate (DBP) is one of widely used synthetic phthalic diesters added to plastics to make them softer. It is used in the making of adhesives, dyes, lacquers, and personal care products. Since DBP is not bound to the final product, through its production and incorporation into products, DBP can be released into the environment. Therefore, DBP has become ubiquitous in the environment, resulting in human exposure [1, 2]. DBP is a potential endocrine disruptor, especially acting on male reproductive system. A case-control study of 176 Chinese infertile men in Taiwan showed the inverse relationship of urine phthalate metabolite levels with Leydig cell function [3]. A cohort study with 501 males in USA also showed the inverse association of urine phthalate metabolites with semen quality [4]. Rodent models demonstrated that DBP can leach out from polyvinyl chloride plastics, disrupting androgen production [5]. DBP was reported to disrupt germ cell development [6], disturb testis development [7], block Leydig cell steroidogenesis [8, 9], and cause Leydig cell abnormal aggregation [10]. These studies indicate that DBP is an endocrine disruptor of male reproduction.

**Figure 1:**
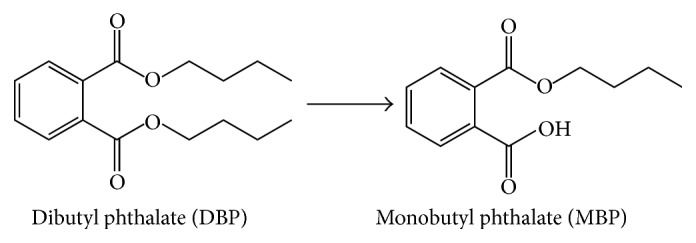
Structures of dibutyl phthalate and monobutyl phthalate and hydrolysis.

Structurally, DBP is a diester. It has been demonstrated that the diester forms of phthalates are rapidly hydrolyzed by esterases in the gut, liver, and blood and are present in the body in monoester forms, which are considered the bioactive toxicants. For example, the monoester form of another phthalate called di(2-ethylhexyl) phthalate (DEHP), mono(2-ethylhexyl) phthalate (MEHP), is reported to be 10-fold more potent in its toxicity to Leydig cells and Sertoli cells compared to DEHP [11]. In this regard, DBP is also metabolized into monobutyl phthalate (MBP) in the body and exists in the monophthalate form (Figure 1). However, the potencies of DBP and MBP to disrupt Leydig cell function as well as the possible mechanism have not been compared.

The puberty is the most sensitive period, during which Leydig cell development has been demonstrated to be disturbed by phthalates [11]. Leydig cells are the steroidogenic cells located in the interstitium of the testis and they produce mainly androgen, which is responsible for onset and maintenance of spermatogenesis and the second characteristics of males. During the puberty, stem Leydig cells exit quiescently, rapidly amplifying the cell number and differentiating into the Leydig cell lineage [12]. During development of Leydig cells in the rat, stem Leydig cells undergo transitions from immature Leydig cells around postnatal day 35 before these cells become mature [13, 14]. The immature Leydig cell is a very unique cell that produces predominantly 5*α*-androstane-3*α*,17*β*-diol (DIOL), because it contains both testosterone biosynthetic and metabolizing enzymes [14]. The testosterone biosynthesis starts substrate cholesterol. The testosterone biosynthetic enzymes include mitochondrial P450 cholesterol side chain cleavage enzyme (CYP11A1, encoded by* Cyp11a1*) and smooth endoplasmic reticulum enzymes 3*β*-hydroxysteroid dehydrogenase 1 (HSD3B1, encoded by* Hsd3b1*), P450 17*α*-hydroxylase/20-lyase (CYP17A1, encoded by* Cyp17a1*), and 17*β*-hydroxysteroid dehydrogenase 3 (HSD17B3, encoded by* Hsd17b3*). Immature Leydig cells express high levels of smooth endoplasmic reticulum steroid 5*α*-reductase 1 (SRD5A1, encoded by* Srd5a1*) and cytosolic 3*α*-hydroxysteroid dehydrogenase (AKR1C14, encoded by* Akr1c14*). The testosterone via biosynthesis in immature Leydig cells undergoes metabolism into dihydrotestosterone (DHT) by SRD5A1 and further into DIOL by AKR1C14 (Supplementary Figure 1 in Supplementary Material available online at http://dx.doi.org/10.1155/2016/1376526) [14].

The androgen production in Leydig cells require a regulatory signal, which is achieved by pituitary-secreted luteinizing hormone (LH). LH binds to its LH receptor (LHCGR, encoded by* Lhcgr*) on the surface of immature Leydig cells, causing the elevation of intracellular cAMP [15]. The LHCGR-cAMP signaling cascade causes the increased expression of scavenger receptor class B member 1 (SCARB1, encoded by* Scarb1*), which helps transporting extracellular cholesterol via low-density lipoprotein, and steroidogenic acute regulatory protein (StAR, encoded by* Star*), which transports cytosolic cholesterol into the inner membrane, where CYP11A1 is located. In the present study, we also investigated the effects of both DBP and MBP on the expression levels of these genes.

## 2. Materials and Methods

### 2.1. Chemicals and Animals

[^3^H]Pregnenolone, [^3^H]progesterone, [^3^H]androstenedione, [^3^H]testosterone, and [^3^H]dihydrotestosterone were purchased from DuPont-New England Nuclear (Boston, MA). Unlabeled pregnenolone, progesterone, 17*α*-hydroxyprogesterone, androstenedione, and testosterone were obtained from Steraloids (Newport, RI). Dibutyl phthalate and monobutyl phthalate were purchased from Sigma (St. Louis, MO). Male Sprague Dawley rats (30-day-old) were purchased from Shanghai Animal Center (Shanghai, China). All animal procedures were approved by the Institutional Animal Care and Use Committee of Wenzhou Medical University and were performed in accordance with the Guide for the Care and Use of Laboratory Animals.

### 2.2. Immature Leydig Cell Isolation

After adjustment for five days, eighteen 35-day-old male Sprague Dawley rats were sacrificed by asphyxiation with CO_2_. Testes were removed and Leydig cells were purified as described previously [14]. In brief, animals were sacrificed in CO_2_ tank, testes were removed, perfused with collagenase (0.1 mg/mL) via testicular artery, digested with collagenase (0.25 mg/mL) and DNase (0.25 mg/mL) for 15 min, and filtered with nylon mesh, and the cells were separated under Percoll gradient. The cells with density of 1.070–1.088 g/mL were collected and washed. Purities of Leydig cell fractions were evaluated by histochemical staining for HSD3B1 activity, with 0.4 mM etiocholanolone as the steroid substrate [16]. The purities of Leydig cells were around 95% consistently.

### 2.3. Leydig Cell Culture

After isolation, the purified immature Leydig cells were seeded into 24-well culture plated with cell density of 0.05 × 10^6^ cells/well. Leydig cells were cultured in 0.5 mL DMEM:F12 medium (phenol-free) without (basal) or with hormone and signaling substances, 10 ng/mL LH and 10 mM 8-bromo-cAMP (8Br-cAMP), 20 *μ*M of various steroid substrates including 22R-OH-cholesterol (22R-OHC), pregnenolone, progesterone, androstenedione, testosterone, and dihydrotestosterone for 3 h in the presence of 0.05–50 *μ*M DBP or MBP (DBP or MBP was dissolved in ethanol and ethanol was the control). Because 8Br-cAMP can penetrate the cell membrane, therefore it is used to replace the intracellular cAMP, which is impermeable. 22R-OHC, pregnenolone, progesterone, androstenedione, testosterone, and dihydrotestosterone were used as the respective substrate of the enzymes CYP11A1, HSD3B1, CYP17A1, HSD17B3, SRD5A1, and AKR1C14. Because 22R-OHC can readily penetrate cell and mitochondrial membrane, it is used to replace cholesterol as substrate for CYP11A1. Media were collected for DIOL and testosterone assay after incubation.

### 2.4. Preparation of Mitochondrial, Cytosol, and Microsomal Proteins

Mitochondrial, cytosol, and microsomal preparations of rat testes were done as described previously [17]. Testes (from 35-day-old Sprague Dawley male rats) were homogenized in cold 0.01 mM phosphate buffered saline (PBS) containing 0.25 mM sucrose and centrifuged at 700 ×g for 30 min. The supernatants were transferred to new tubes and centrifuged at 10,000 ×g for another 30 min and washed twice to collect mitochondrial pellet. Supernatants then were further centrifuged at 105,000 ×g for 1 h twice to collect microsomal pellet and supernatant as cytosol. Pellets were resuspended and protein concentrations in these fractions were measured using the Bio-Rad Protein Assay Kit (cat# 500-0006, Bio-Rad, Hercules, CA) according to manufacturer's protocol. Mitochondria were used for CYP11A1 measurement. Microsomes were used for measurement of CYP17A1 and SRD5A1 enzyme activities. Cytosol was used for AKR1C14 measurement.

### 2.5. CYP11A1 Assay

CYP11A1 activity in testicular mitochondria was assayed using 22R-OHC as a substrate and pregnenolone as a product. Briefly, 22R-OHC (20 *μ*M) was dissolved in ethanol, with final ethanol concentration in the reaction solution no more than 0.2%. In assays to determine the inhibitory potencies of DBP and MBP, concentrations of 22R-OHC at 20 *μ*M were added to reaction mixture containing 10 *μ*g rat testis mitochondria and 50 *μ*M DBP and MBP, and the mixtures were incubated at 34°C for 3 h. By end of incubation, the product, pregnenolone, was assayed by RIA kit. The percentage conversion of 22R-OHC into pregnenolone was calculated by pregnenolone from the substrate.

### 2.6. Enzymatic Assays of CYP17A1, SRD5A1, and AKR1C14

The testicular microsomal and cytosol enzymatic assays were done as described previously [18]. The detailed conditions for each enzymatic assay were listed as follows: briefly, the mixture (250 *μ*L) of the substrates (0.2 *μ*M P4 for CYP17A1, 0.2 *μ*M T for SRD5A1, and 1 *μ*M DHT for AKR1C14), the tracers (40,000 dpm), and cofactors (NADPH, 0.2 mM, for all these enzymes) were incubated with certain amounts of enzymes (microsomal fractions for CYP17A1 and SRD5A1 or cytosol fraction for AKR1C14) for 60–90 min at 34°C (the temperature of normal testis). For some reactions, DBP and MBP were added as inhibitors (up to 50 *μ*M). By the end of incubations, the reactions were stopped by adding 2 mL ice cold ether. The steroids were extracted, and the organic layer was dried under nitrogen. Steroids were separated chromatographically on thin layer plates (Baker-Flex Silica Gel IB-F coated with 200 *μ*m analytical layer and fluorescent indicator, 20 × 20 cm, Thomas Scientific, Swedesboro, NJ) in chloroform-ether (7 : 1, v/v) for CYP17A1 assay or chloroform-methanol (97 : 3, v/v) for SRD5A1 or diethyl ether-acetone (98 : 2, v/v) for the AKR1C14 assay. The radioactivity was measured using a scanning radiometer (System AR2000, Bioscan Inc., Washington, DC). The percentage conversion of the substrates into products was calculated by dividing the radioactive counts identified as products by the total counts of substrates plus products.

### 2.7. Assay of Pregnenolone, DIOL, and Testosterone Concentrations

Pregnenolone, DIOL, and testosterone concentrations in the medium were measured with a tritium-based radioimmunoassay as described [14], using the commercial RIA kits (IBL, USA). Interassay variation of the pregnenolone, DIOL, and testosterone was within 15%.

### 2.8. Extraction of RNA and Real Time PCR (qPCR)

Total RNAs were extracted from immature Leydig cells using Trizol reagent (Invitrogen, Carlsbad, CA, USA) according to the manufacturer's instruction. Twelve Leydig cell genes and their primers were used as described previously [19, 20]. These genes are membrane receptor genes including* Lhcgr*, cholesterol transporting genes including* Scarb1* and* Star*, and steroidogenic enzyme genes, including CYP11A1 (*Cyp11a1*), HSD3B1 (*Hsd3b1*), CYP17A1 (*Cyp17a1*), HSD17B3 (*Hsd17b3*), SRD5A1 (*Srd5a1*), and AKR1C14 (*Akr1c14*), as well as steroidogenesis-regulatory transcription factor* Nr5a1*. The cell proliferation genes, including* Pcna* and* Ccnd1*, were also included. The relative mRNA levels of targeted genes were normalized to* Rps16* (internal control gene). The RNA was reversely transcribed into cDNA using random hexamers and MMLV reverse transcriptase by the kit (Promega, CA) according to the manufacturer's instruction. qPCR was carried out in a 25-*μ*L reaction volume with SYBR Green detection system (Bio-Rad Laboratories, Inc., Hercules, CA, USA). Reactions were run on a Bio-Rad qPCR system (Bio-Rad Laboratories, Inc., Hercules, CA, USA) for up to 40 cycles and the melting curves were always checked afterward.

### 2.9. Statistics

Data were subjected to analysis by Student's *t*-test to identify significant differences whenever two groups (a single concentration of DBP or MBP versus control) were compared. Data were subjected to analysis by Kruskal-Wallis test followed by ad hoc Dunnett's multiple comparisons to identify significant differences between the tested group and the control whenever three or more groups (multiple concentrations of DBP or MBP versus control) were compared. All experiments were repeated 3–5 times, depending on the experiments. All data are expressed as means ± SEM. Differences were regarded as significant at *P* < 0.05.

## 3. Results

### 3.1. Effects of DBP and MBP on Androgen Production in Rat Immature Leydig Cells

**Figure 2:**
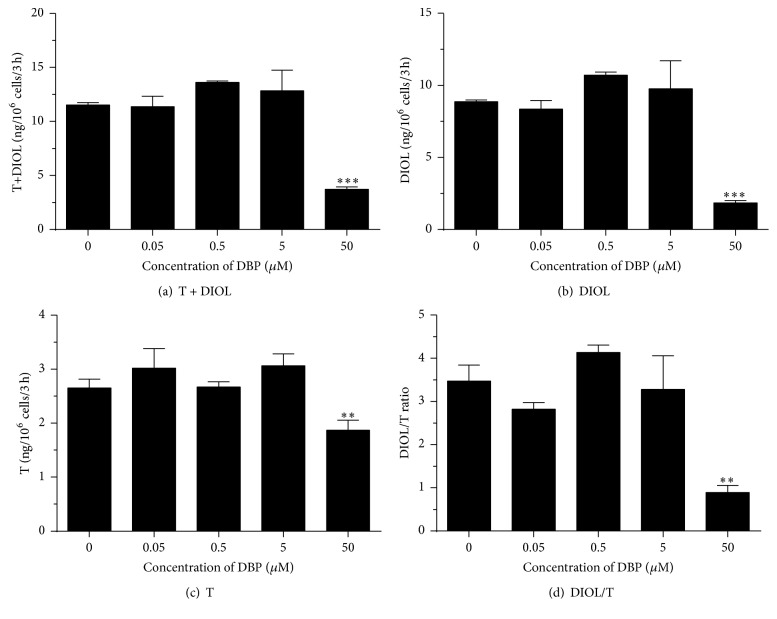
Concentration dependent effects of DBP on basal androgen production by rat immature Leydig cells. Rat immature Leydig cells were cultured with 0.05–50 *μ*M dibutyl phthalate (DBP) for 3 hrs. Medium testosterone (T) and 5*α*-androstanediol (DIOL) levels were measured. (a) T + DIOL; (b) DIOL; (c) T; and (d) DIOL/T ratio. Mean ± SEM, *n* = 4; *∗*, *∗∗*, and *∗∗∗* indicate significant difference when compared to control at *P* < 0.05, 0.01, and 0.001, respectively.

**Figure 3:**
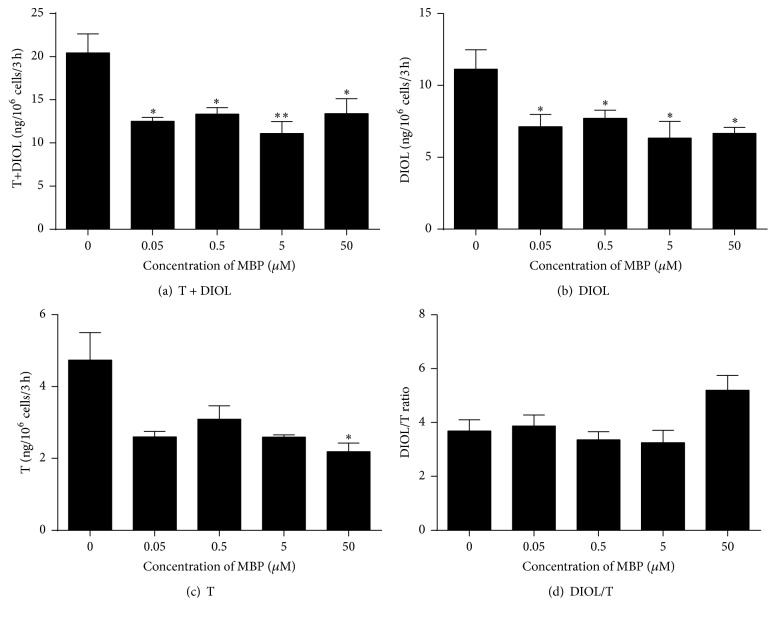
Concentration dependent effects of MBP on basal androgen production by rat immature Leydig cells. Rat immature Leydig cells were cultured with 0.05–50 *μ*M monophthalate (MBP) for 3 hrs. Medium testosterone (T) and 5*α*-androstanediol (DIOL) levels were measured. (a) T + DIOL; (b) DIOL; (c) T; and (D) DIOL/T ratio. Mean ± SEM, *n* = 4; *∗*, *∗∗*, and *∗∗∗* indicate significant difference when compared to control at *P* < 0.05, 0.01, and 0.001, respectively.

The rat immature Leydig cell primarily produces DIOL, because it contains androgen metabolizing enzymes (SRD5A1 and AKR1C14) [14] (Supplementary Figure 1). We tested the effects of DBP (Figure 2) and MBP (Figure 3) on androgen biosynthesis and metabolism. As shown in Figure 2, at the highest concentration (50 *μ*M) tested, DBP significantly inhibited total androgen (T + DIOL, Figure 2(a)), DIOL (Figure 2(b)), and T (Figure 2(c)) productions, indicating that DBP can inhibit androgen biosynthesis at this concentration. The ratio of DIOL to T (Figure 2(d)) was significantly reduced by 50 *μ*M DBP, indicating that androgen metabolism is also blocked at this concentration. MBP from the lowest concentration (50 nM) to the highest concentration (5 *μ*M) inhibited total androgen (Figure 3(a)) and DIOL (Figure 3(b)) levels. Only at 50 *μ*M did MBP inhibit T production (Figure 3(c)). The ratio of DIOL to T was not changed (Figure 3(d)), indicating that MBP does not influence androgen metabolism. This indicates that MBP is more potent than parent compound DBP to inhibit androgen production.

**Figure 4:**
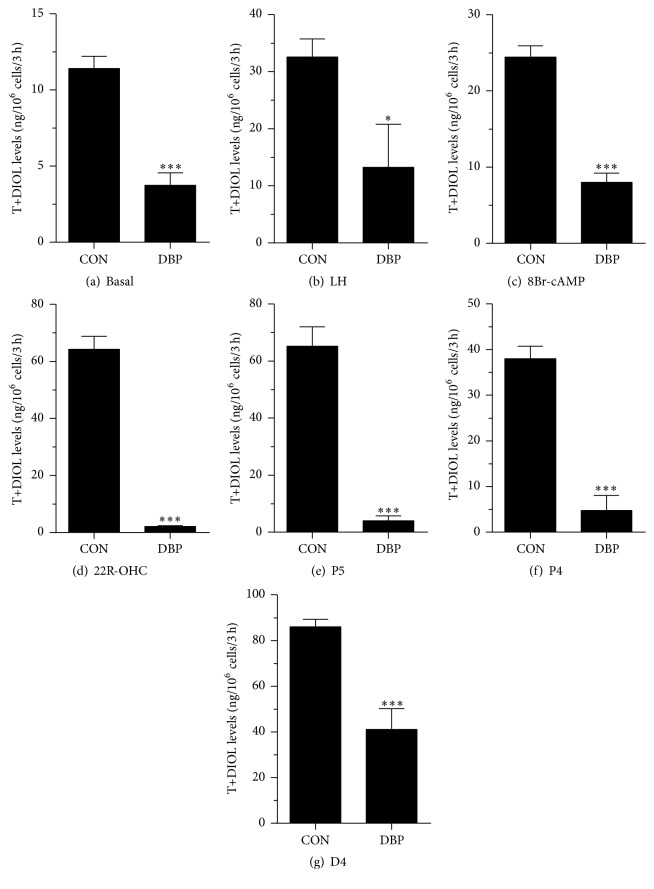
Effects of DBP on androgen production by rat immature Leydig cells. Rat immature Leydig cells were cultured without or with luteinizing hormone (LH), 8bromo-cAMP (8Br-cAMP), 22R-OH-cholesterol (22R-OHC), pregnenolone (P5), progesterone (P4), and androstenedione (D4) in combination with 50 *μ*M DBP for 3 hrs. Medium 5*α*-androstanediol (DIOL) and testosterone (T) levels were measured. Mean ± SEM, *n* = 4; *∗*, *∗∗*, and *∗∗∗* indicate significant difference when compared to control at *P* < 0.05, 0.01, and 0.001, respectively.

We further compared the effects of DBP and MBP on androgen production and metabolism of rat immature Leydig cells using 50 *μ*M concentration of each phthalate. We added hormone (LH, 10 ng/mL), signaling compound (8Br-cAMP, 10 mM), and steroidogenic enzyme substrates, including those of CYP11A1 (22R-OHC, 20 *μ*M), HSD3B1 (pregnenolone, P5, 20 *μ*M), CYP17A1 (progesterone, P4, 20 *μ*M), HSD17B3 (androstenedione, D4, 20 *μ*M), SRD5A1 (testosterone, T, 20 *μ*M), and AKR1C14 (DHT, 20 *μ*M), and measured the medium DIOL and T and then compared the total androgen (T + DIOL) with the control (no treatment, basal). As shown in Figure 4 (DBP), and as expected, under basal, LH, and 8Br-cAMP stimulations, DIOL was the major androgen, which accounted for 9-fold over T level (Table 1). At basal, LH, and 8Br-cAMP stimulated conditions, DBP consistently inhibited T + DIOL productions. Because the inhibitions were comparable between LH and 8Br-cAMP stimulations, it is suggested that the inhibition site(s) may be beyond the LH signaling cascade. After addition of 22R-OHC, P5, P4, and D4 as substrates, the final androgen output (T + DIOL) was all barely detectable for 22R-OHC, P5, and P4 but increased a little bit toward controls when D4 was used as a substrate (Figure 4(g)). It suggests that the major inhibition is between the cascades from cholesterol to androstenedione (CYP11A1, HSD3B1, and CYP17A1).

**Figure 5:**
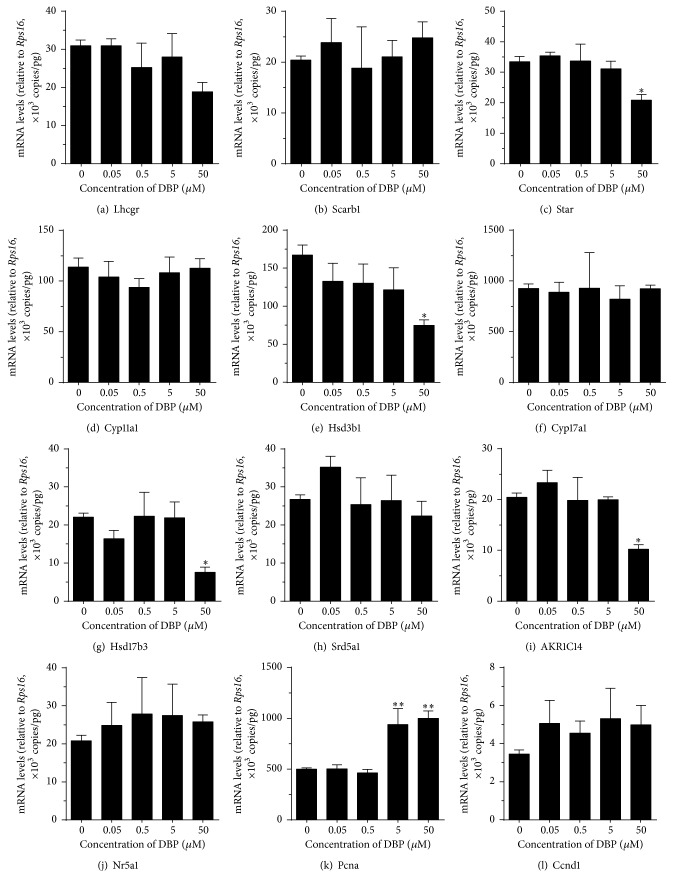
Effects of DBP on the expression levels of steroidogenesis related genes in rat immature Leydig cells. Rat immature Leydig cells were cultured with 0.05–50 *μ*M DBP for 3 hrs. The expression levels of steroidogenesis related genes were measured and calculated relatively to* Rps16*, the internal control. Mean ± SEM, *n* = 4; *∗*, *∗∗*, and *∗∗∗* indicate significant difference when compared to control at *P* < 0.05, 0.01, and 0.001, respectively.

Interestingly, MBP did not inhibit LH- and 8BR-cAMP stimulated total androgen levels though it inhibited basal androgen level (Figure 5). After addition of 22R-OHC, P5, P4, and D4 as substrates, the final androgen outputs (T + DIOL) were not affected either by MBP, indicating that MBP does not affect CYP11A1, HSD3B1, CYP17A1, and HSD17B3 at this concentration.

When T and DIOL were considered separately (Table 1), the results were also intriguing. DBP caused remarkable reduction of DIOL levels at basal, LH, 8Br-cAMP, 22R-OHC, P5, and P4 additions. With D4 as a substrate, T production was not affected (Table 1), while the production of DIOL was significantly reduced (Table 1). This suggests that, in addition to the T synthetic cascades, the T metabolizing cascades (SRD5A1 and AKR1C14) were also affected by DBP. Since there was a minimal reduction for DIOL, when T was used as the substrate, while there was a remarkable reduction when DHT was used as the substrate, it is possible that AKR1C14, not SRD5A1, is affected by DBP. For MBP, there were no dramatic effects for all conditions (Table 1), indicating that at this concentration MBP does not affect androgen biosynthetic and metabolizing enzymes.

### 3.2. Concentration Dependent Effects of DBP and MBP on the Expression Levels of Genes Related to Androgen Biosynthesis

We examined the effects of DBP on the expression levels of genes that are related to androgen biosynthesis and metabolism (Figure 5). Statistically, we found that at 50 *μ*M DBP significantly downregulated the* Star*,* Hsd3b1*,* Hsd17b3*, and* Akr1c14* levels (Figure 5). The downregulation of* Star* level indicates that the rate-limiting step of cholesterol transportation from cytosol into inner membrane of mitochondrion is disrupted by DBP. The downregulation of* Hsd3b1*,* Hsd17b3*, and* Akr1c14* levels also confirmed the inhibitions of DBP on their respective enzymes.

**Figure 6:**
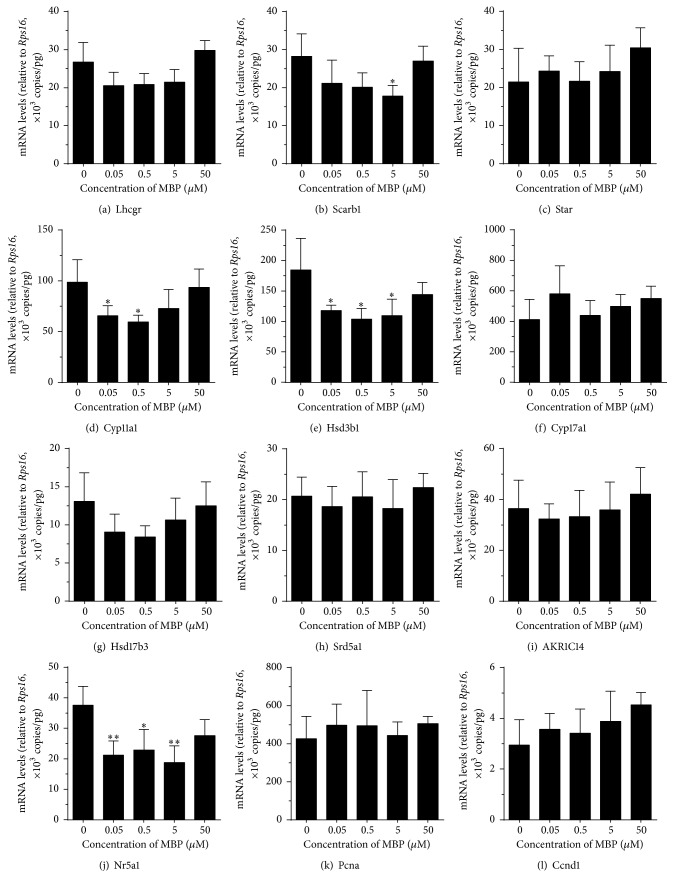
Effects of MBP on the expression levels of steroidogenesis related genes in rat immature Leydig cells. Rat immature Leydig cells were cultured with 0.05–50 *μ*M MBP for 3 hrs. The expression levels of steroidogenesis related genes were measured and calculated relatively to* Rps16*, the internal control. Mean ± SEM, *n* = 4; *∗*, *∗∗*, and *∗∗∗* indicate significant difference when compared to control at *P* < 0.05, 0.01, and 0.001, respectively.

We also examined the effects of MBP on the expression levels of genes that are related to androgen biosynthesis and metabolism (Figure 6). Statistically, we found that at as low as 50 and 500 nM MBP significantly downregulated the* Cyp11a1* and* Hsd3b1* levels (Figure 6) and at 5 *μ*M it inhibited* Scarb1* level. Surprisingly, at 50 *μ*M none of the genes tested were affected. Interestingly, the level of transcription factor* Nr5a1*, which is critical for the regulation of expressions of* Cyp11a1* and* Hsd3b1*, was significantly reduced by 50 nM–5 *μ*M MBP, corresponding to the downregulation of its target genes (*Cyp11a1* and* Hsd3b1*).

### 3.3. The Direct Inhibition on Androgen Biosynthetic and Metabolizing Enzyme Activities by DBP and MBP

**Figure 7:**
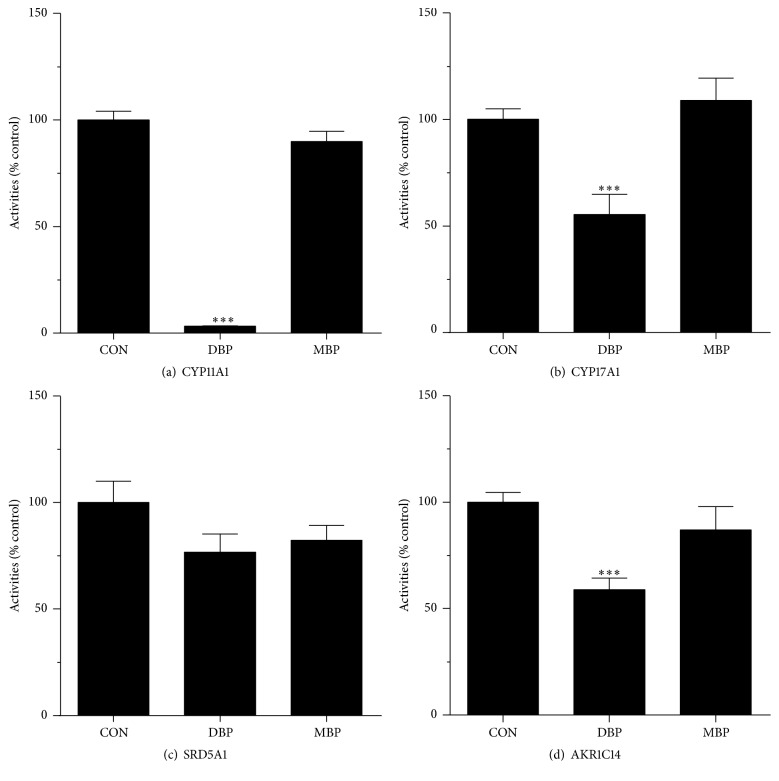
Direct effects of DBP and MBP on androgen biosynthetic and metabolizing enzyme activities in rat testes. Rat testis enzymes were measured with 50 *μ*M DBP and MBP. The % activity of control was calculated. Mean ± SEM, *n* = 4; *∗*, *∗∗*, and *∗∗∗* indicate significant difference when compared to control at *P* < 0.05, 0.01, and 0.001, respectively.

There was discrepancy between DBP-mediated effects on some steroidogenic enzyme expression levels and androgen production. At 50 *μ*M DBP did not affect* Cyp11a1* and* Cyp17a1* levels but caused almost barely detectable 22R-OHC and P4-mediated androgen levels. We previously demonstrated that DBP directly inhibited rat HSD3B1 activity and marginally inhibited rat HSD17B3 activity, while MBP did not inhibit these enzymes at all [21]. We tested whether DBP and MBP also directly inhibited other androgen biosynthetic (CYP11A1, CYP17A1) and metabolizing enzyme (SRD5A1 and AKR1C14) activities. As shown in Figure 7, at 50 *μ*M DBP significantly inhibited CYP11A1, CYP17A1, and AKR1C14 activities, while it had no effect on SRD5A1 activity. MBP, at 50 *μ*M, had no effects on all these enzyme activities. This indicates that the reduction of 22R-OHC and P4-mediated androgen production in DBP-treated cells is caused by its direct inhibition on these enzyme activities.

## 4. Discussion

In this study, the purified immature Leydig cell has shown different sensitivity to endocrine disruptor DBP and its metabolite MBP. In the* in vitro* culture environment, only high concentration (50 *μ*M) of DBP showed significant inhibition on the production of both testosterone (T) and DIOL. On the other hand, from low concentration (50 nM) to high concentration (5000 nM), MBP showed significant inhibition on androgen production.

When DBP enters the mammalian body, it is rapidly hydrolyzed by esterases in the gut, liver, and blood into MBP. It seems that DBP and MBP showed different potencies and mechanism to suppress steroidogenesis. Generally speaking, the metabolite MBP was more potent to inhibit androgen production (Figure 3). At as low as 50 nM, MBP significantly inhibited androgen production (Figure 3). This inhibition is mostly contributed by the downregulation of some steroidogenesis related genes,* Cyp11a1* and* Hsd3b1*. However, MBP did not block completely the transcription of these genes. The residual expression levels of these two genes were about 60%. Thus, the residual activities of CYP11A1 and HSD3B1 were about 60% after 50–5000 nM MBP treatment (Figure 3). However, MBP did not directly inhibit androgen biosynthetic and metabolizing enzyme activities when up to 50 *μ*M was used. Therefore, the MBP-induced inhibition is most likely from its suppression of these gene expressions.* Cyp11a1* and* Hsd3b1* are the target genes of transcription factor NR5A1 (encoded by* Nr5a1*). The expression levels of* Cyp11a1* and* Hsd3b1* also followed the pattern of* Nr5a1* after MBP treatment (Figure 6(j)). Interestingly, 50 *μ*M MBP did not interfere with steroidogenesis related gene expressions, indicating that higher concentration of MBP loses its action. We did not test even higher concentrations of MBP. However, our and other studies with another monophthalate, MEHP, also showed an even stimulatory action when higher concentrations were used.

Interestingly, the potency of DBP to inhibit androgen production was less than that of MBP. However, at 50 *μ*M, DBP can cause significant inhibition of androgen production. This indicates that DBP was 1000-fold less potent than MBP. However, at 50 *μ*M, DBP caused more deep inhibition of androgen production. The residual levels of androgen after DBP treatment at basal, LH, 8Br-cAMP, 22R-OHC, P5, P4, and D4 were 33%, 41%, 31%, 3%, 3%, 7%, and 37%, respectively. This level was achieved by DBP-mediated multiple mechanisms, especially via direction inhibition. In this regard, 50 *μ*M DBP induced 3.3% residual levels of CYP11A1 (Figure 7). In our previous study, we also demonstrated that 50 *μ*M DBP also caused about 25% of residual activities of rat HSD3B1 and HSD17B3 [21]. Furthermore, 50 *μ*M DBP also decreased the expression levels of* Hsd3b1* and* Hsd17b3* by 45% and 34%, respectively (Figure 5). Therefore, at this concentration, DBP caused more deep suppression of androgen production.

Inhibitions in these key biosynthetic enzymes surely could contribute to the inhibition of testosterone production by DBP and MBP, but the diversity of enzyme and gene expression inhibition ability indicate different mechanism of inhibition between DBP and MBP. Apparently, the inhibitions in synthetic pathway may play more dominant role than that in the metabolic process because, even in the face of reductions in metabolic process, testosterone was still reduced.

Our previous study demonstrated that rat immature Leydig cells showed some mitosis ability [22]. In the present study, we found that DBP but not MBP promoted* Pcna* expression. PCNA (proliferating cell nuclear antigen) is a biomarker of Leydig cell proliferation [23–25]. Increased* Pcna* expression levels indicate that DBP induces the proliferation of rat immature Leydig cells. Indeed, it was reported that* Pcna* expression levels were also higher in the Leydig cells after* in utero* DBP exposure [26].

MBP is the main metabolite of DBP after oral administration [27]. It has been reported that MBP was the major metabolite in the urine of the children (8 years old) [28], juveniles (12 years old) [29], and adults (32 years old in average) [30]. Due to the wide use as a material for cosmetics, the endocrine disruption of DBP has caused public concern. Indeed, it has been reported that the urinary phthalate metabolite levels were negatively related to the reduced Leydig cell function and poor quality of semen [3, 4].

In conclusion, DBP and its metabolite MBP showed different potencies to inhibit androgen production in rat immature Leydig cells. MBP was about 1000-fold more potent than its parent compound DBP. At lower concentrations, MBP inhibited androgen production mostly via the downregulation of* Cyp11a1* and* Hsd3b1* expression levels. At the higher concentration (50 *μ*M), DBP inhibited androgen production mostly via direct inhibition of CYP11A1, HSD3B1, and HSD17B3 activities or downregulating* Star* and* Hsd3b1* expression levels.

## Supplementary Material

The steroidogenic pathway in rat immature Leydig cells will be shown in the supplementary material. Cholesterol is transported into Leydig cells via lipoprotein (LP)-receptor (SCARB1). When LH binds to luteinizing hormone receptor (LHCGR) to induce LHCGR-complex interaction leading to cAMP cascade, the latter activates protein kinase A, which induces the expression of steroidogenic acute regulatory protein (STAR). STAR is the rate-limiting step to transport intracellular cholesterol into mitochondrial inner membrane.

## Figures and Tables

**Table 1:** The effects of dibutyl phthalate (DBP) and monobutyl phthalate (MBP) on androgen production in rat immature Leydig cells.

	Androstanediol (ng/10^6^ cells/3 h)			Testosterone (ng/10^6^ cells/3 h)		
	Control	DBP	MBP	Control	DBP	MBP
Basal	9.80 ± 0.64^a*∗*^	1.91 ± 0.19^b^	7.20 ± 0.28^c^	3.68 ± 0.47^a*∗*^	1.83 ± 0.27^b^	2.43 ± 0.20^b^
LH	27.17 ± 1.50^a^	4.68 ± 0.66^b^	19.18 ± 1.05^c^	6.67 ± 0.73^a^	1.58 ± 0.27^b^	7.67 ± 1.26^a^
8BR-cAMP	19.47 ± 0.59^a^	6.46 ± 0.44^b^	11.27 ± 0.21^c^	4.99 ± 0.85^a^	1.56 ± 0.18^b^	5.29 ± 0.62^a^
22R-OH-cholesterol	51.43 ± 2.63^a^	2.06 ± 0.11^b^	41.14 ± 0.89^c^	12.02 ± 0.84^a^	0.06 ± 0.01^b^	8.62 ± 0.014^a^
Pregnenolone	48.26 ± 2.55^a^	3.51 ± 0.85^b^	42.39 ± 5.29^a^	16.85 ± 1.32^a^	0.55 ± 0.09^b^	16.70 ± 1.27^a^
Progesterone	23.71 ± 1.38^a^	4.04 ± 1.26^b^	27.07 ± 2.96^a^	14.31 ± 0.30^a^	2.60 ± 1.89^b^	17.92 ± 1.37^a^
Androstenedione	71.50 ± 1.39^a^	30.13 ± 4.06^b^	74.01 ± 0.39^a^	14.50 ± 0.37^a^	11.92 ± 1.18^a^	19.10 ± 1.25^b^
Testosterone	92.37 ± 0.58^a^	82.90 ± 2.17^b^	85.56 ± 1.34^b^	ND	ND	ND
Dihydrotestosterone	184.8 ± 1.15^a^	91.19 ± 2.37^b^	182.4 ± 1.34^a^	ND	ND	ND

Mean ± SEM, *n* = 4~12. ND = not detected. ^*∗*^Identical letters indicate that there are no significant differences between two groups for either androstanediol or testosterone production.
