# Supplementary material for: Comparison of the Effects of Dibutyl and Monobutyl Phthalates on the Steroidogenesis of Rat Immature Leydig Cells
Source: Biomed Res Int. 2016 Apr 11;2016:1376526. doi: 10.1155/2016/1376526 (PMC4842363; doi:10.1155/2016/1376526)

**Supplementary Fig1. Steroidogenic pathway in rat immature Leydig cells**

Cholesterol is transported into Leydig cells via lipoprotein (LP)-receptor (SCARB1). When LH binds to luteinizing hormone receptor (LHCGR) to induce LHCGR-complex interaction leading to cAMP cascade, the latter activates protein kinase A, which induces the expression of steroidogenic acute regulatory protein (STAR). STAR is the rate-limiting step to transport intracellular cholesterol into mitochondrial inner membrane. P450 cholesterol side chain cleavage enzyme (CYP11A1); 3β-hydroxysteroid dehydrogenase 1 (HSD3B1); 17α-hydroxylase/C17,20-lyase (CYP17A1); 17β-hydroxysteroid dehydrogenase 3 (HSD17B3); 5α-reductase 1 (SRD5A1); 3α-hydroxysteroid dehydrogenase (AKR1C14).


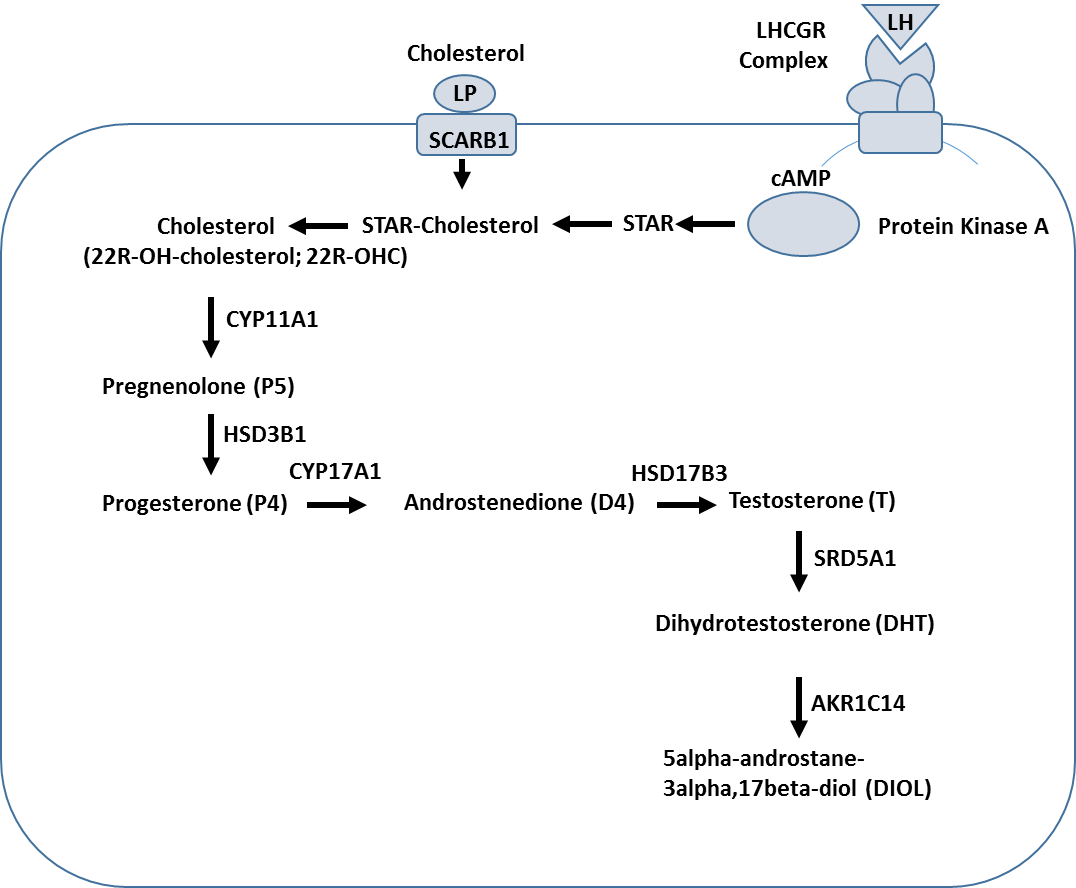

Supplement: Supplementary file 1 — The steroidogenic pathway in rat immature Leydig cells will be shown in the supplementary material. Cholesterol is transported into Leydig cells via lipoprotein (LP)-receptor (SCARB1). When LH binds to luteinizing hormone receptor (LHCGR) to induce LHCGR-complex interaction leading to cAMP cascade, the latter activates protein kinase A, which induces the expression of steroidogenic acute regulatory protein (STAR). STAR is the rate-limiting step to transport intracellular cholesterol into mitochondrial inner membrane. [file 1376526.f1.docx]
